# A novel attention-getting vocalization in zoo-housed western gorillas

**DOI:** 10.1371/journal.pone.0271871

**Published:** 2022-08-10

**Authors:** Roberta Salmi, Monica Szczupider, Jodi Carrigan

**Affiliations:** 1 Department of Anthropology, University of Georgia, Athens, GA, United States of America; 2 Intergrative Conservation Graduate Program, University of Georgia, Athens, GA, United States of America; 3 Zoo Atlanta, Atlanta, Georgia, United States of America; University of Birmingham, UNITED KINGDOM

## Abstract

As a critical aspect of language, vocal learning is extremely rare in animals, having only been described in a few distantly related species. New evidence, however, extends vocal learning/innovation to the primate order, with zoo-housed chimpanzees and orangutans producing novel vocal signals to attract the attention of familiar human caregivers. If the ability to produce novel vocalizations as a means of navigating evolutionarily novel circumstances spans the Hominidae family, then we can expect to find evidence for it in the family’s third genus, *Gorilla*. To explore this possibility, we conduct an experiment with eight gorillas from Zoo Atlanta to examine whether they use species-atypical vocalizations to get the attention of humans across three different conditions: just a human, just food, or a human holding food. Additionally, we survey gorilla keepers from other AZA-member zoos to compile a list of common attention-getting signals used by the gorillas in their care. Our experiment results indicated that Zoo Atlanta gorillas vocalized most often during the human-food condition, with the most frequently used vocal signal being a species-atypical sound somewhere between a sneeze and a cough (n = 28). This previously undescribed sound is acoustically different from other calls commonly produced during feeding (i.e., single grunts and food-associated calls). Our survey and analyses of recordings from other zoos confirmed that this novel attention-getting sound is not unique to Zoo Atlanta, although further work should be done to better determine the extent and patterns of transmission and/or potential independent innovation of this sound across captive gorilla populations. These findings represent one of the few pieces of evidence of spontaneous novel vocal production in non-enculturated individuals of this species, supporting the inclusion of great apes as moderate vocal learners and perhaps demonstrating an evolutionary function to a flexible vocal repertoire.

## Introduction

Language is considered a uniquely human feature, though most of its components—such as vocal learning, intentionality, syntax, semantics, and other associated cognitive abilities—varyingly emerge in the communication systems of other animals [1, 2]. Yet despite the mechanical similarities between vocal production and perception in human and non-human primates (hereafter primates) [3] as well as our shared evolutionary history, some of these cognitive features seem to be absent from the communication systems of our closest relatives. Indeed, evidence for “complex vocal learning,” or the ability to produce novel calls through the imitation of sounds, is rare in the animal kingdom [4, 5], confirmed only in three bird orders (songbirds, parrots, and hummingbirds: [6, 7]), some cetacean [8–10], bat [11, 12], and pinniped species [13, 14], and only recently in elephants [15, 16]. Recently developed frameworks for studying vocal learning, however, have departed from distinguishing vocal from non-vocal learners based only on the presence/absence of vocal mimicry alone, and consider the ability to fine-tune the acoustic structure of species-specific vocal signals in the absence of auditory input as evidence of vocal learning [17–20].

Research has long classified primate vocalizations as innate signals not modifiable by experience [21] and as driven only by the internal state [22, 23]. New evidence, however, demonstrates otherwise: call structural plasticity [24], call convergence [25–27], turn-taking exchanges [28–30], and reinforcement-based vocal learning during development [31] all suggest that primates may at least be limited or moderate vocal learners [18, 32]. Moreover, research examining audience effect on primate communication suggests that some primates have volitional control on vocal production [33] as well as an awareness of the receiver’s perceptual state, potentially indicating some aspects of theory of mind [34, 35]. For instance, female vervet monkeys produce more alarm calls when offspring are nearby [36], male Thomas langurs stop calling only after all group members reply with a counter call [37], while chimpanzees produce exaggerated screams if nearby group members can provide support [38] and more frequent alarm calls if bystanders are unaware of imminent danger [39–41]. Audience effect also influences the production of food calls. Capuchin monkeys delay calling if other individuals are far away [42], while chimpanzees call more often when food quantity is sharable [43] or if estrous females are nearby [44]. Multiple zoo-based experiments have indicated that great apes can adjust their vocal strategies when they are tested across different conditions: specifically, (1) in the presence of visible but inaccessible food, (2) in the presence of an inaccessible and inattentive human without food, or (3) in the presence of an inaccessible and inattentive human with food. These experiments have confirmed that great apes reliably modify their responses according to the perceived attentional state of human experimenters: in other words, they show greater efforts at getting the attention of humans during the third condition [45–49], presumably since the humans could access foods that the apes themselves could not. Moreover, these apes attuned their strategies if they were not intially successful, demonstrating increased effectiveness at communication [50].

Evidence of vocal learning and/or innovation, although scant, is slowly accumulating for captive apes: orangutans can learn to produce voiced utterances [51–53] and whistles [54, 55], chimpanzees adopt new referential food calls through vocal convergence under social integration [56], and enculturated apes, such as the gorilla Koko and the chimpanzee Vicky, are able to produce a limited number of novel utterances [57, 58]. Most of these novel signals seem to emerge while communicating with human caregivers. For instance, Hopkins, Taglialatela [59] explored species-atypical attention-getting vocal strategies in chimpanzees, behaviorally and acoustically describing the “raspberry” and the extended grunt, which they explicitly classified as “novel signals invented in novel environmental circumstances” (p281). Wich, Swartz [54] showed that orangutans are able to imitate human whistling to attract and direct human attention, an ability later confirmed in at least ten other captive orangutans [55]. Furthermore, growing evidence suggests the novel call types emerging in captive settings can indeed be socially learned (chimpanzees: [60–62]; orangutans: [55]).

Despite an emerging trajectory and a growing need to catalog novel vocal signals produced in captivity, particularly those signals used to get the attention of humans, little has been reported for gorillas. Here we test whether gorillas use species-atypical vocal signals to attract the attention of humans, as reported for both orangutans and chimpanzees. We compare the vocal and gestural signals produced by the gorillas during three conditions: 1) *only keeper*, where a keeper sits in front of the subject’s cage, 2) *only food*, where a bucket of food is positioned in front of the subject’s cage, and 3) *keeper holding food*, where a keeper holds a bucket of food in front of the subject’s cage. We hypothesize that if gorillas use vocal and/or gestural signals to capture a human’s attention, they will use them more often during the third condition than during the first two [46]. In identifying atypical vocal signals, we provide the acoustic description and compare them to the species-specific calls that are most frequently uttered while feeding: contact calls [grunts: 63] and food-associated calls [hums: 63, 64]. Finally, we present the results of a survey of 39 gorilla zookeepers across 19 North American AZA-member zoos to assess the types of attention-getting signals used in captivity by this species.

## Methods

### Study subjects

**Fig 1:**
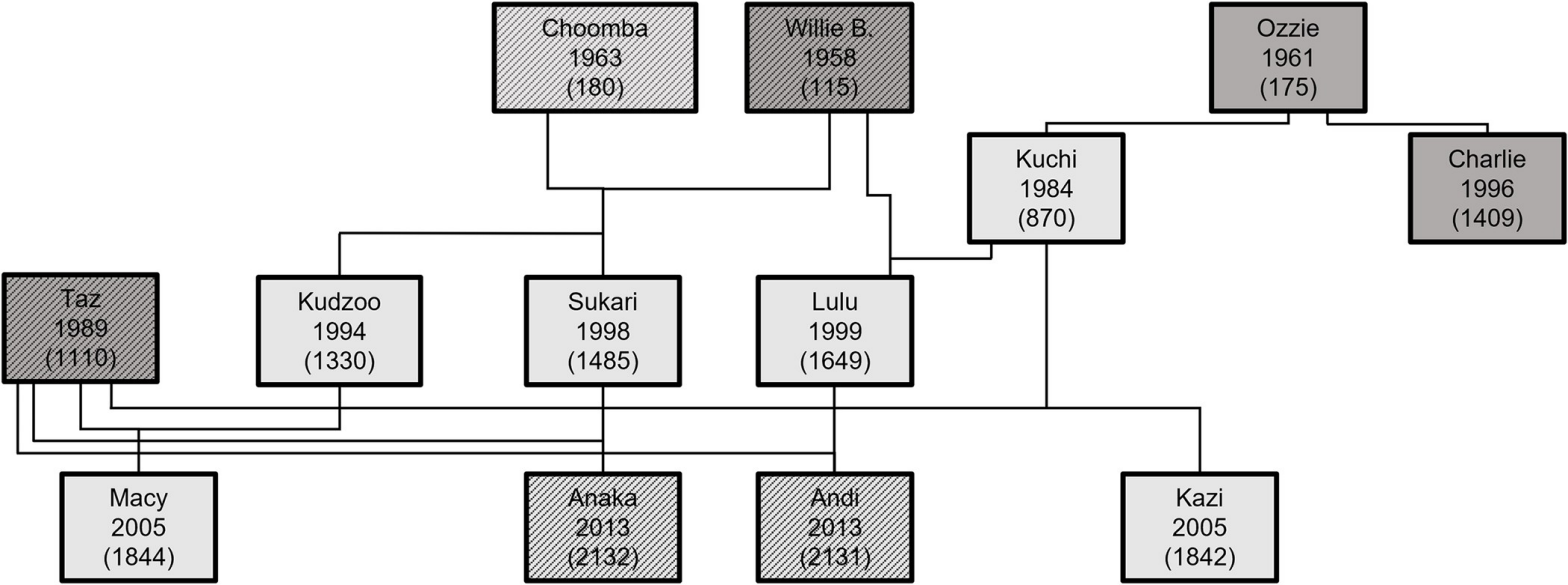
Kinship, year of birth, and studbook ID of study subjects. Light and dark gray boxes indicate female and male gorillas, respectively, and patterned background indicate gorillas not included in the experimental study but included here to indicate kinship.

We included eight gorillas (*Gorilla gorilla gorilla*), 2 males (19–55 years old) and 6 females (9–31 years old), in the experimental portion of the study. We conducted the experiments at Zoo Atlanta between April and May 2015. We indicate kinship, date of birth, and Studbook ID numbers for each subject in Fig 1. We included all the female gorillas from Taz’s group as well as two external adult males whose testing did not interfere with the keepers’ work plans during the days of data collection.

### Testing procedure

We tested each subject in his/her indoor cage either in isolation or in pairs, and mothers always in the company of dependent infants. At all times subjects were able to see, smell, and communicate vocally with group members and other gorillas occupying the same indoor enclosure (even though they were in separate chambers during the experiment). The keeper staff generally followed a similar procedure of separating gorillas during feeding sessions to reduce competition between group members and to ensure that all individuals received an adequate amount of food; thus, no disruption of daily gorilla routines occurred while conducting this study. Our research protocol was approved by the Zoo Atlanta Scientific Review and by the Committee Institutional Animal Care and Use Committee of the University of Georgia and complies with the ASAB/ABS guidelines for the Use of Animals in Research.

We tested the subjects once per three conditions: *only keeper* (K), *only food* (F), and *keeper holding food* (KF). During each condition, we positioned the keeper and/or food out of reach and in clear sight of the gorilla subject at 1 meter from their enclosure. In the first condition (K), the keeper sat on a stool facing sideways (body and face turned 90° from the subject). In the second condition (F), the keeper placed fresh grapes in a bucket on a stool, tilting the bucket to ensure its contents were in complete sight of the subject. The keeper vacated the area just before the start of the experiment. Finally, in the third condition (KF), the keeper sat on a stool facing sideways (body and face turned 90° from the subject) holding a tilted bucket of grapes to ensure the subject could see its content. The keeper conducting the experiments has worked with the Zoo Atlanta gorillas for over 15 years, currently serves as the Assistant Curator of Primates, and is one of the present study’s co-authors (JC). We tested each subject consecutively in all three conditions, with an interval of 1 min between conditions. We randomized the sequences of the conditions across subjects. Each experiment lasted 120 seconds, and we recorded the results with a Panasonic HC-VX870 4K Ultra HD Camcorder and/or a Canon Powershot G12 with a built-in microphone. To ensure high quality recordings of vocal behavior during the experiments, we also positioned a Sennheiser MKH 416 short shotgun microphone at 1–1.5 m from the subject’s cage, protecting it with a foam windshield (MZW415ANT) and connecting it to a Professional Solid State Recorder Marantz PMD671. Additionally, because the acoustic quality of recordings was variable and not many calls were produced during the experiments, we complemented the vocal sample with *ad libitum* acoustic recordings of single grunts, hums, and attention-getting sounds (the same calls produced during the experiments) from 7 Zoo Atlanta gorillas (including all of the females that participated in the experiments, plus another female, Shamba) during 26 days of data collection between January and October 2015, using the same equipment described above.

MS coded the videos, categorizing attention-getting strategies as either vocalizations (distinguishing types: e.g., attention-getting call, grunts, grumbles) or gestures. We further categorized gestures as either auditory (e.g., handclapping, chest-beating, or enclosure-banging) or non-auditory (e.g., tool-use, attempting to touch oneself or another gorilla, or reaching fingers through the mesh toward the food and/or keeper). To test for coding reliability, MS recoded twenty percent of the videos one year later. We used frequencies of occurrence of attention-getting sounds, other vocalizations, and gestures to assess intra- and inter-rater reliability using a two-way mixed, absolute agreement, intra-class correlation [65]. The resulting intra-class correlation coefficients (ICC) were all in optimal range [>0.90; 65], indicating high intra-rater consistency: test-retest correlation for attention-getting sounds was 0.98, for other vocalizations was 0.96, and for gestures 1.00. We repeated the same procedure but for different raters after the experimenter (RS) recoded twenty percent of the videos and obtained again significant and high correlations for each signal type considered (1.00, 0.93, and 0.94 respectively).

### Acoustic analysis

We made all digital recordings at a sampling frequency of 48 kHz and saved them as uncompressed.WAV files (PCM format). To facilitate the measurements of acoustic and temporal parameters, we converted the sampling frequency of all calls to 11,025 Hz, using the Avisoft SASLab Pro software (R. Specht, Berlin, Germany). We generated spectrograms using a frequency resolution of 1024 points and the Hamming Window option. We selected only good quality recordings (low background noise-call ratio) for a total of 64 calls. Gorilla vocalizations can be produced in isolation, in series [63], or in combination [66]. We considered the unit of analysis the single segment (since the three call types were never combined with other calls during the experiments) and measured for each call one temporal parameter, the call duration, and 4 frequency parameters, the mean frequency 1^st^ dfa (distribution of frequency amplitude), the mean frequency 2^nd^ dfa, the minimum difference between 1^st^ and 2^nd^ df (dominant frequency bands), and the maximum peak frequency, to broadly describe temporal and spectral call characteristics, using a custom software program [LMA: 67]. In cases where calls were given in bouts (series), we also reported the mean duration of the silent interval between calls. Mean values of call parameters are given as value±SD.

To determine if the calls labeled as attention-getting were acoustically distinct from the other calls produced during the experiments (i.e., single grunts and food calls or hums: [63, 64]), we performed a discriminant function analysis [DFA: 68] and a leave-one-out cross validation DFA using the five acoustic parameters mentioned above [69]. For this analysis we included calls of high quality recorded during the experiments as well as other recordings of the same call types recorded *ad libitum* at another time (as before mentioned). We included calls produced by all 6 of the female gorillas that participated in the experiments plus the grunts of one additional Zoo Atlanta female gorilla, Shamba, to increase the sample size of this call type (see Table 2 in S1 File for each female sample size; and S1 Data for the entire dataset). In addition, to account for non-independence, we ran a permuted DFA (pdfa.incomplete–since not all callers contributed to each call type) using the function written by Roger Mundry in R (version 4.2.0; Core Team 2013), based on the function lda of the MASS R Package [70]. The procedure includes 100 random selections and 1000 iterations, allows to control for caller ID, and provides the statistical significance (equivalent to p-value) of the mean effect size of cross-validated classification. We then assessed whether the parameters significantly associated with the DFA functions (r > 0.5) were statistically useful to discriminate across call types using linear mixed models [LMM: 71] when controlling for repeated (Caller ID as random factor) and unequal sampling among individuals (Satterthwaite approximation). We adjusted for multiple analyses using Sequential Bonferroni method. With the exception of the pDFA described above, all statistical analyses were conducted using SPSS 25.0 (IBM Corp., NY USA).

### Attention-getting and other novel sounds in other zoos

To obtain a robust sampling of attention-getting behaviors used by zoo gorillas toward their human caregivers, we created a fourteen-question survey in Qualtrics (S1 Appendix) and distributed it to 47 AZA member zoos housing gorillas in the United States and Canada. We used both closed-ended questions (those requiring a yes or no answer or those where respondents were asked to list the ages and sexes of gorillas) as well as open-ended questions (those where respondents were asked to qualitatively describe a particular behavior). We asked respondents to meet the following conditions in order to take part in the survey: (1) to have at least one year of experience working directly with the gorillas on whom they reported and (2) to be able to recognize the individual gorillas on sight. We also requested at least two individual survey responses from each zoo, but this request was not a requirement.

## Results

### Experiments

The Zoo Atlanta gorillas produced three types of vocalizations during the experiments, listed here in order of frequency: a novel call type indicated here as an attention-getting sound (AG) (29), single grunts (3), and hums (2) (Table 1). Interestingly, only half of the individuals we tested produced the novel attention-getting sound (Table 1), and they were all adult female gorillas. Three of them are closely related: Sukari and Kudzoo are sisters, and Macy is Kudzoo’s first daughter (see Fig 1). The attention-getting gestures produced during the experiments included those that were auditory (i.e., gestures executed in order to produce a distinct audible component, such as hand-clapping, chest-beating, or enclosure-banging) and those that were non-auditory (i.e., gestures that did not invariably produce a distinct audible component, such as tool-use, attempting to touch oneself or another gorilla, or reaching fingers through the mesh toward the food and/or keeper). Table 2 Column B lists all attention-getting strategies exhibited by Zoo Atlanta gorillas.

**Table 1:** Number of signals for each individual in the three conditions.

Subject	S	A	Attention-Getting Calls			Other Vocalizations			Gestures		
			*F*	*K*	*KF*	*F*	*K*	*KF*	*F*	*K*	*KF*
Kudzoo	F	21	0	0	7	0	0	0	0 (0)	0 (0)	2(1)
Kuchi	F	30	0	0	2	0	0	1	0 (0)	0 (0)	7 (0)
Lulu	F	15	0	0	0	0	0	0	0 (0)	0 (0)	11 (9)
Sukari	F	16	0	0	7	0	0	2	0 (0)	0 (0)	2 (0)
Macy	F	9	0	1	12	0	0	0	1 (0)	5 (1)	26 (4)
Kazi	F	9	0	0	0	0	0	0	4 (0)	0 (0)	2 (0)
Charlie	M	19	0	0	0	0	0	1	1 (0)	0 (0)	1 (0)
Ozzie	M	54	0	0	0	0	0	1	0 (0)	0 (0)	12 (12)

S = sex; A = age in 2015; F = food; K = keeper; KF = keeper with food. Note: Total gestures include those that are non-auditory and auditory. Of the total gestures, we indicated the # of auditory gestures in parentheses.

**Table 2:** Attention-getting behaviors reported by survey respondents from other zoos vs. attention-getting behaviors documented at Zoo Atlanta. N indicates the number of individuals displaying the behaviors. Note: For N (other zoos), we count responses only once where multiple respondents from the same zoo described the same call for the same individual.

Description	Column A	Column B
*We provide descriptions of calls or gestures as they have been previously documented*. *Where a vocalization or gesture was reported by only one survey respondent*, *we report that respondent’s description*.	**N**	**N**
	*(Other zoos)*	*(Zoo Atlanta)*
**Attention-getting vocalizations**		
*Attention-getting call*: Per respondent, a call where the lips are puckered, mouth is open and one to three short vocalizations are produced	>5<33*	4
*Grumble/rumble/purr/hum*: A low, long call of irregular length and dense harmonics bearing varying degrees of noise and produced by an open or closed mouth [63]	27	3
*Raspberries*: A voiceless call where the lips buzz or sputter to produce the sound [57, 98]	11	0
*Bark*: A loud, harsh, and abrupt call most often produced as a single call but occasionally in repetition by adult males [63]	7	0
*Kiss/squeak/lip smack*: A voiceless, ingressive sound produced by the lips [22, 94]	5	0
*Grunt*: A soft, guttural single- or double-syllable call with noisy harmonics, produced with an open or closed mouth at a low frequency [65]	4	5
*Cry/scream*: A call with high, sparse harmonics, varying degrees of noise, and produced in a sequence of varying lengths [65]	3	0
*Burp*: Per respondent, also referred to as a “frog burp” and used by the gorilla to startle people	1	0
*Unspecified vocalization*: No further description provided by respondent	1	0
**Attention-getting gestures, auditory**		
*Banging/knocking/hitting/tapping the mesh/enclosure/window/wall/door*	27	3
*Hand clapping*	15	1
*Chest beating*	3	1
**Attention-getting gestures, non-auditory**		
*Shaking/waving/tapping/lifting part of body*	5	3
*Pushing objects through mesh*, *trading for object*, *using tools to reach object*	8	0
*Staring*	3	0
*Following keeper*	2	0
*Soliciting play*	1	0
*Throwing hay*	1	0
*Presenting/pushing body against mesh (4); biting mesh (4)*	0	8
*Reaching toward mesh (4)*, *grabbing (1)*	0	5
*Displaying*	0	1
**Unknown** (not clear if accompanied by auditory signal)		
*Flipping enrichment feeder (1); refusing to comply with keeper instructions (1)*	2	0

*NOTE: Through the analysis of videos and recordings of 15 gorillas from other zoos, we were able to confirm the use of the target call in 6 gorillas. The number of gorillas reported to use the target call from keepers totaled 33: Calgary Zoo (2), Columbus Zoo and Aquarium (2), Dallas Zoo (5), Houston Zoo (1), North Carolina Zoo (1), Oklahoma City Zoo (1), Riverbanks Zoo and Garden (3), Utah’s Hogle Zoo (1), WCS Bronx Zoo (5), Woodland Park Zoo (11), Zoo Knoxville (1).

**Fig 2:**
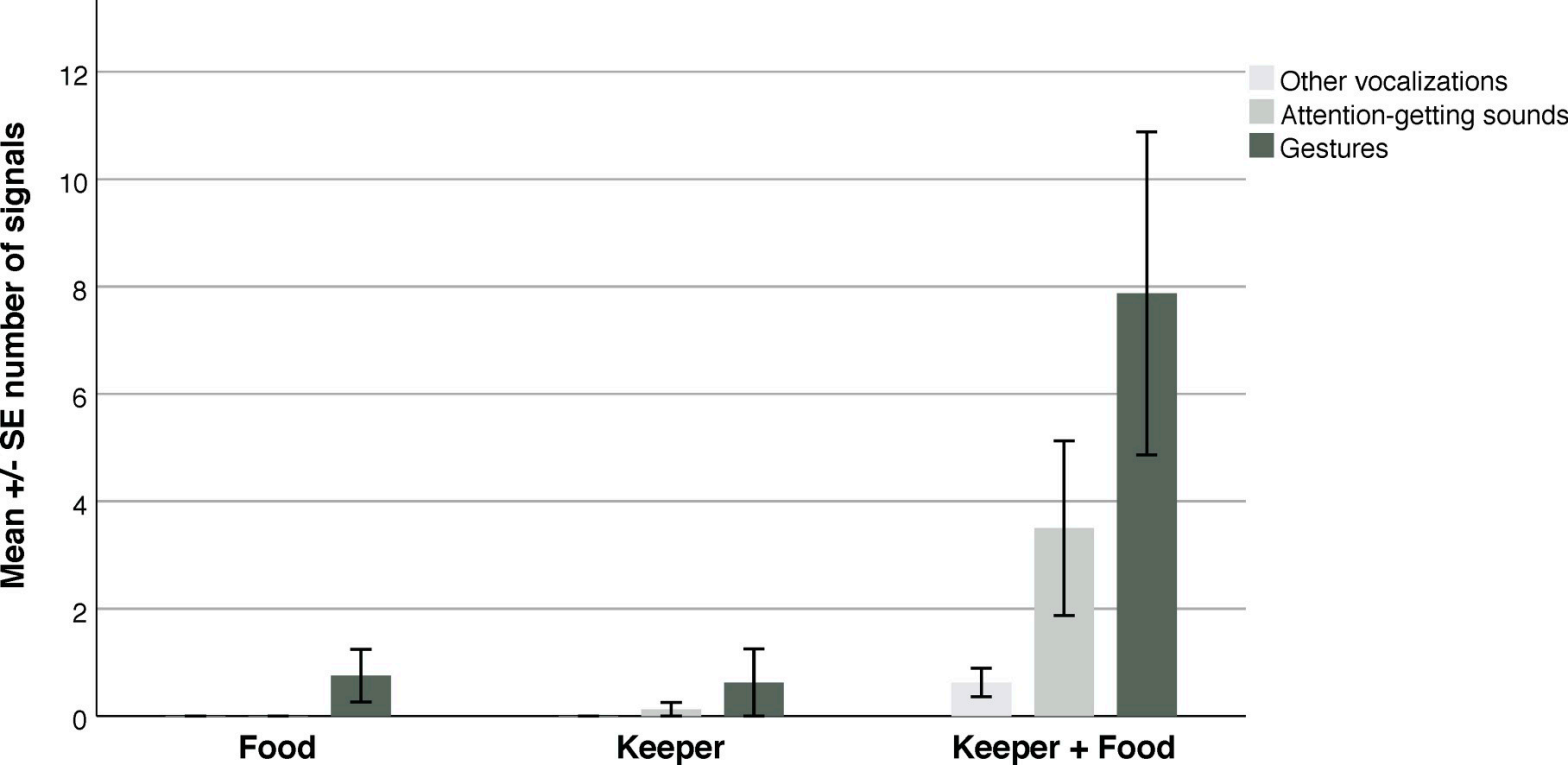
Mean number of vocalizations, attention-getting sounds, and gestures in each condition. Error bars represent standard errors; the three signal types differed significantly between conditions (VOC: p = 0.018; AG: p = 0.023; and GES: p = 0.006).

The frequency of attention-getting sounds (AG), other vocalizations (VOC), and gestures (GES) differed significantly between experimental conditions (Friedman test: AG: χ^2^ = 7.54; p = 0.023; VOC: χ^2^ = 8.00; p = 0.018; and GES: χ^2^ = 10.23; p = 0.006; auditory and non-auditory gestures did not differ significantly and thus are analyzed together (Z = -1.29 p = 0.65)—when analyzed separately they both differed between conditions: auditory gestures: χ^2^ = 7.54; p = 0.02, non-auditory gestures: χ^2^ = 8.44; p = 0.015). The gorillas produced all three types of signals more often during the KF condition than compared to either the F or K condition alone (Fig 2). However, the small sample size (N = 8) prevented us from finding significant pairwise comparisons when adjusting for multiple analyses with the Bonferroni method, except for gestures being produced significantly more often during the KF condition than the K condition (Wilcoxon rank test: K vs. KF: z = -2.53; p < 0.025).

### Attention-getting call

**Fig 3:**
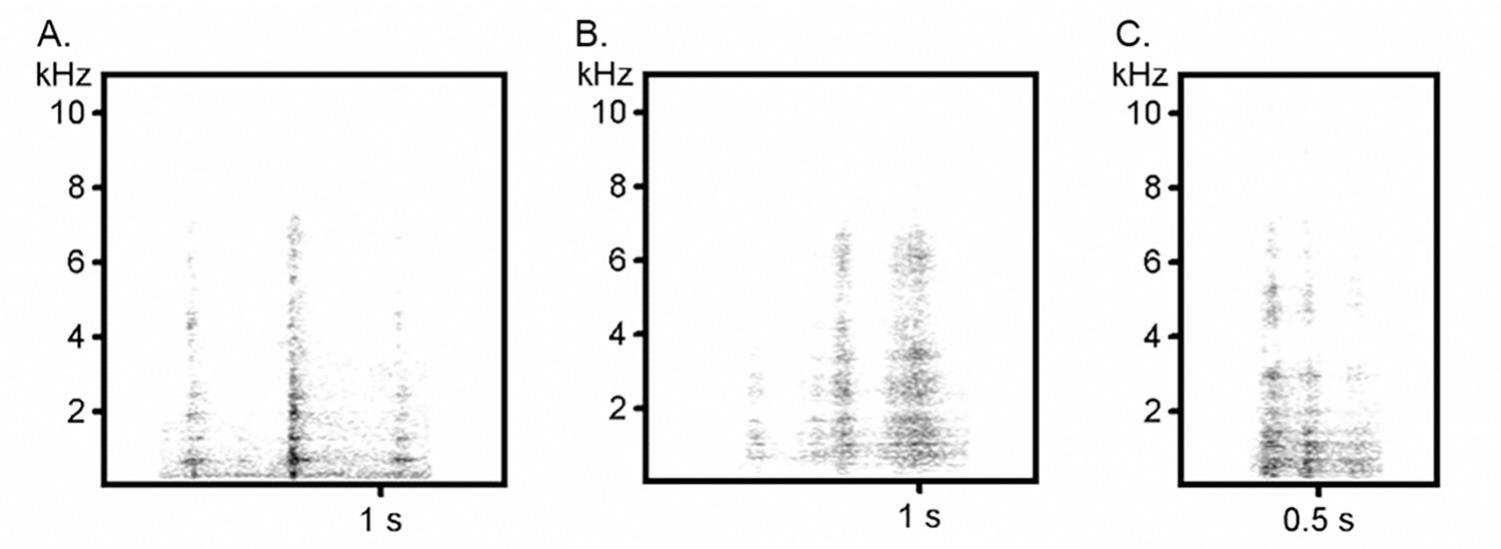
Attention-getting call bouts from three study subjects: Kudzoo (A), Macy (B), and Sukari (C). The y-axis displays frequencies (kHz) and the x-axis time (seconds).

Captive gorilla attention-getting calls resemble a sound between a sneeze and a cough that we named “snough” or AG (video clip in S1 Video). The gorillas at Zoo Atlanta generally produced these signals as single calls, but in a few cases, they were part of a longer series with 2 to 4 calls spaced on average by 0.8±1.2 s (min = 0.09 s, max = 2.5 s; see Fig 3 for bout samples of three females). The mean duration of the single call was 212.4±63 ms, with Macy and her mother Kudzoo having shorter calls than those of the other two adult females. On average, the mean frequencies of the 1^st^ and 2^nd^ dfa (distribution of frequency amplitude) were 553±281 Hz and 1110±43 Hz, respectively. The minimum difference in frequency between the 1^st^ and 2^nd^ dominant frequency bands was in average below 200 Hz (195.5±43.0), while the maximum peak frequency was almost 1000 Hz (997.6±357.6) (see Table 3). The calls produced by the three related females (Sukari, Kudzoo, and Macy) were often accompanied by an exaggerated mouth opening and/or a gentle but fast repeated slapping/covering of head or face.

**Table 3:** Individual acoustic measurements of attention-getting calls, or “snough", for each female. Listed are the following: duration of single element; duration of silent interval between calls (number of intervals considered) when given in bouts; mean frequency 1^st^ dfa (distribution of frequency amplitude) (Q1mean); mean frequency 2^nd^ dfa (Q2mean); Minimum difference between 1^st^ and 2^nd^ df (dominant frequency bands) (Diffmin); and Maximum peak frequency (Pfmax).

Subject	Call n	Duration (ms)	Intercall interval (s)	Q1mean	Q2mean	Diffmin	Pfmax
				(Hz)	(Hz)	(Hz)	(Hz)
Sukari	18	233.0±100.0	0.15±0.1 (n = 20)	365.2±103.8	762.3±270.0	171.9±44.6	642.9±295.5
Kuchi	3	288.1± 39.5	2.50±1.8 (n = 2)	478.0±209.0	1187.7±532.8	152.0±17.7	990.3±1056.8
Macy	3	141.1±73.9	0.10±0.1 (n = 3)	968.3±88.9	1634.7±222.2	208.3±46.3	1489.0±273.9
Kudzoo	7	187.3±75.0	0.19±0.2 (n = 4)	401.4±186.1	854.3±317.8	249.7±80.1	867.3±760.4
*Female Mean*		*212*.*4±62*.*9*	*0*.*8±1*.*2*	*553*.*2±280*.*7*	*1109*.*8±394*.*8*	*195*.*5±43*.*0*	*997*.*6±357*.*6*

**Fig 4:**
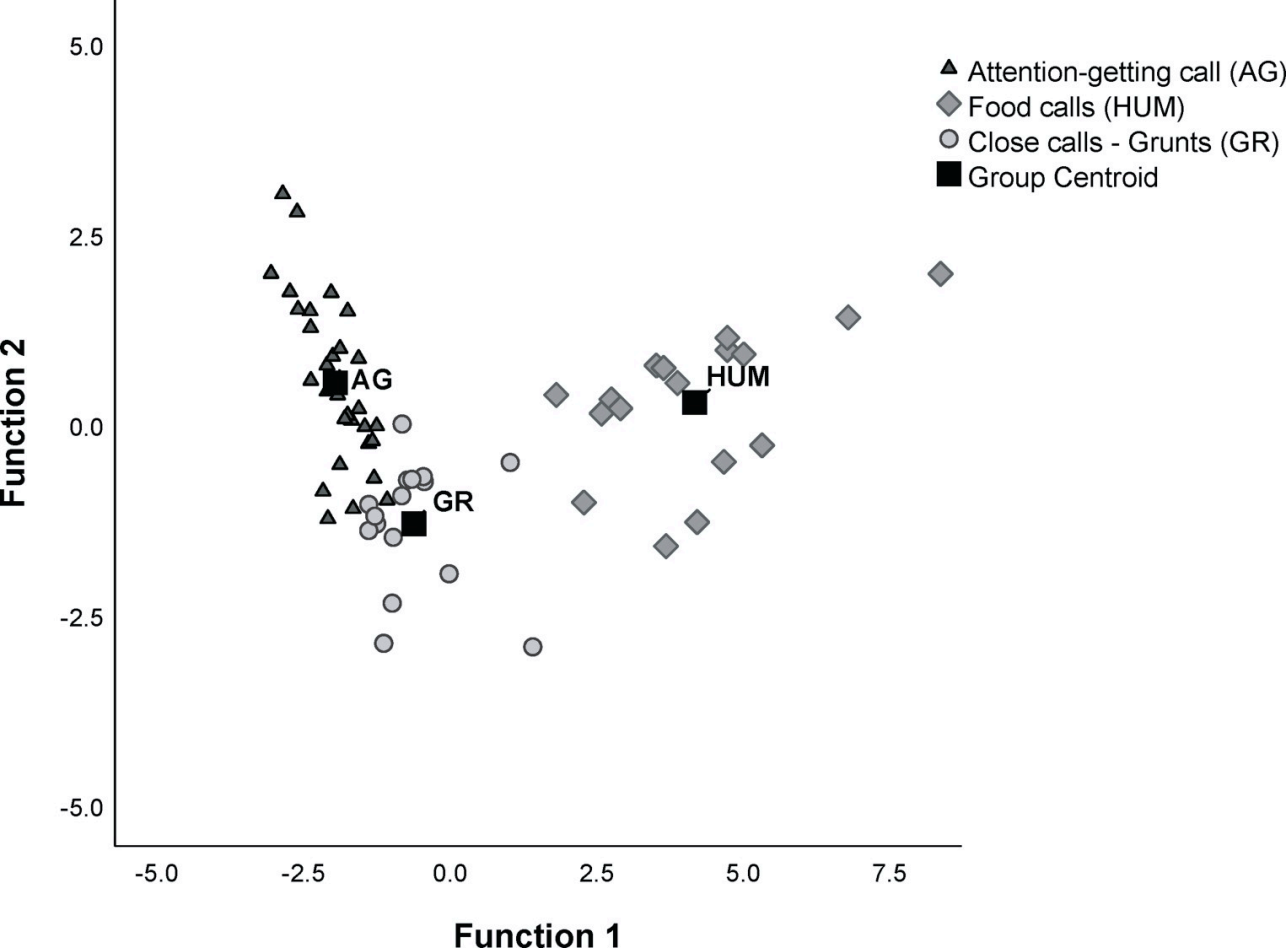
Plot of the two canonical discriminant functions for the three call types in captive western gorilla.

**Fig 5:**
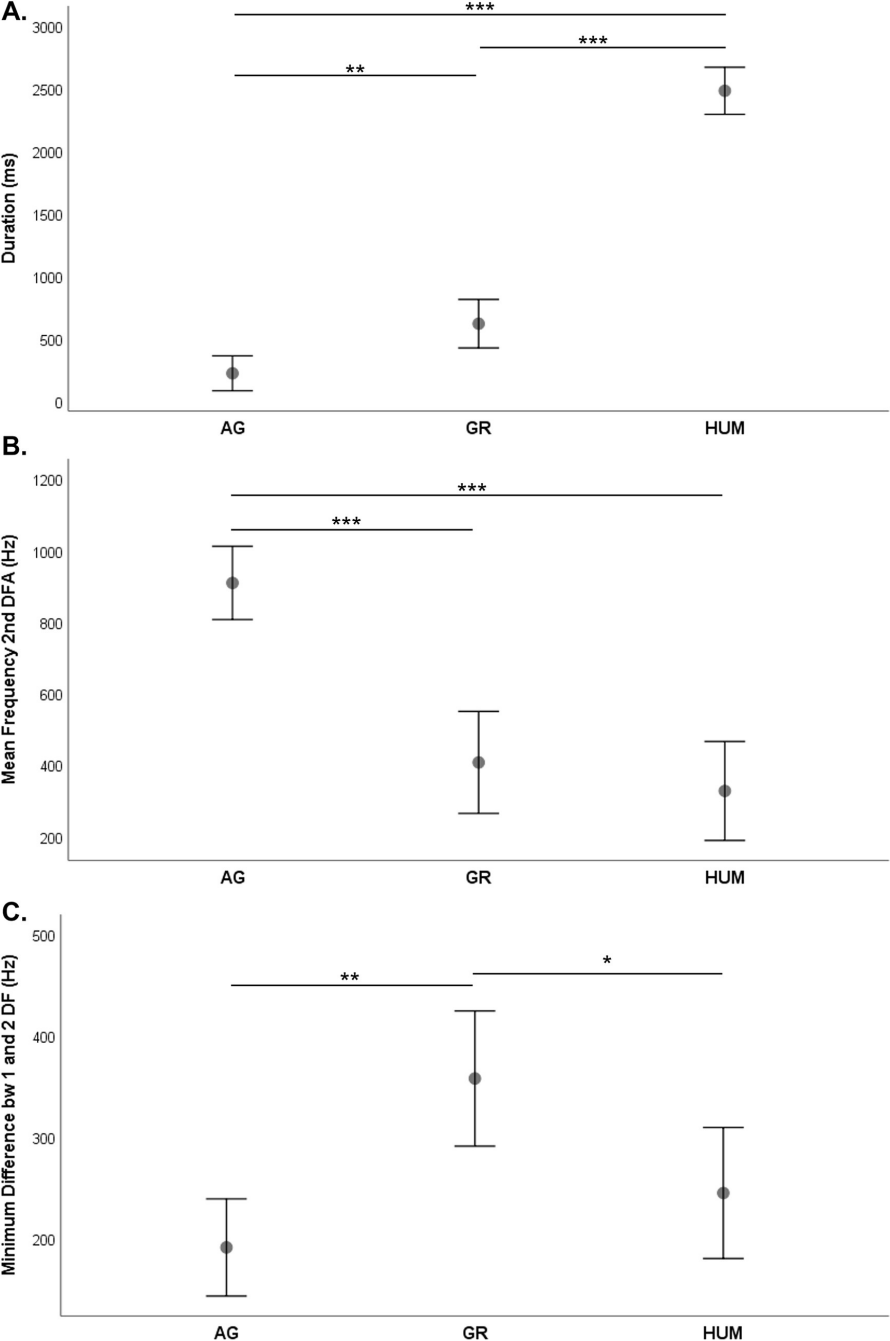
a, b, and c. Pairwise comparisons among call types. Tested for the following: a) duration, b) mean frequency of the 2^nd^ distribution of frequency amplitude (dfa), and c) minimum difference between 1^st^ and 2^nd^ dominant frequency band (df). Asterisks indicate the level of significance: *** p < 0.001, ** p <0.01; * p< 0.05.

To test whether the novel attention-getting call we identify here differs acoustically from common gorilla calls such as grunts and food calls, we ran a discriminant function analysis (DFA). For this analysis, we used call recordings only from the female gorillas since the males never produced the attention-getting calls and since male and female calls may vary greatly due to the large difference in body size [170.4 vs. 71.5 kg; 72]. The DFA distinguished among the three call types, with a classification accuracy of 92% for both the original and cross-validated DFAs (Wilks’ λ = 0.078; χ^2^ = 152.99; df = 6; p <0.001), indicating that the profiles derived from the calls are highly stable [73]. Two canonical discriminant functions were generated: Function 1 explained 91.7% and Function 2 8.3% of the variance. While Function 1 was primarily correlated with the call duration (r = 0.95), Function 2 was correlated with the mean frequency of the 2^nd^ dfa (r = 0.73) and the minimum difference in frequency between the 1^st^ and 2^nd^ dominant frequency bands (r = - 0.65). The percentages of corrected assigned calls varied between 87% (attention-getting calls) to 100% (food calls or hums) (Table 4). Misclassified calls were only recorded between attention-getting (AG) calls and grunts, with 1 grunt classified as AG call and 4 AG calls misclassified as grunts (Fig 4). The permuted DFA (pDFA) indicated that the calls were still significantly different when controlling for caller ID, with 78% of cross-classified calls assigned correctly (p = 0.028). Linear Mixed Model results had confirmed that the three acoustic parameters used in the DFA differed significantly among calls when controlling for caller ID (duration: F_(2, 51)_ = 157.95; p < 0.001; q2mean: F_(2, 58)_ = 31.56; P < 0.001; diffmean: F_(2, 54)_ = 9.34; p< 0.001). While duration differed significantly between each call type pairs, the mean frequency of the 2^nd^ dfa distinguished attention-getting calls from the other two calls, the minimum difference in frequency between 1^st^ and 2^nd^ dominant frequency bands distinguished grunts from attention-getting calls and hums (Fig 5; see S2.1 Table in S1 File for pairwise comparison results).

**Table 4:** Classification table for the cross-validated discriminant function analysis with percentage (and number) of calls assigned to each call type.

		Predicted group membership		
Call Type	N	AG calls	Grunts	Hums
**AG calls**	31	87.1% (27)	12.9 (4)	0
**Grunts**	17	0	100% (17)	0
**Hums**	16	6.3% (1)	0	93.8% (15)

92.2% of original and cross-validated grouped cases correctly classified.

### Attention-getting signals and presence of the novel call across AZA-member zoos

**Fig 6:**
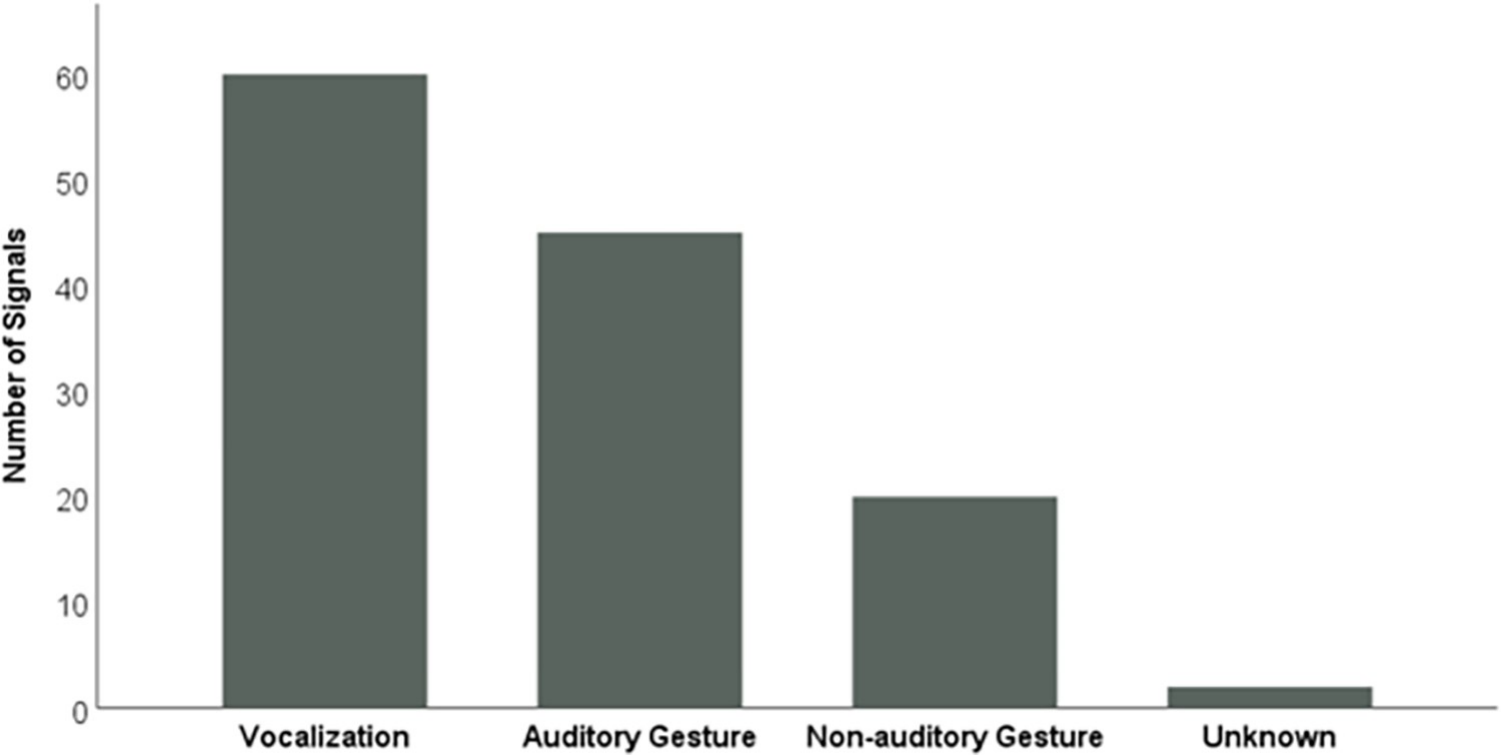
Counts of gorilla attention-getting signals reported by caregivers in 19 facilities. These signals are subdivided by vocalizations, auditory gestures, and non-auditory gestures. Not included here are the Zoo Atlanta gorillas.

Between August 2020 and February 2021, we received 39 individual survey responses from keepers at 19 AZA-member zoos hosting ~118 gorillas, which was roughly a third of the total gorilla population in AZA facilities during that time [74]. All survey respondents reported having previously observed attention-getting strategies directed toward them by one or more gorillas in their care, and 34 respondents (87.2%) from 17 institutions (89.5%) described at least one type of vocalization. Overall, auditory signals accounted for over 84% of all attention-getting strategies reported, including vocalizations (e.g., grumbles, purrs, and raspberries: 47.2%) and auditory gestures (e.g., banging, chest beating, and hand clapping: 35.4%), while non-auditory gestures accounted for only 15.7% of attention-getting strategies reported (Fig 6; Table 2 Column A). Eighteen survey respondents (46.2%) confirmed the presence of the target vocalization in the vocal repertoires of at least one of the gorillas in their care. In total, these respondents attributed the vocalization to 33 gorillas (F = 23, M = 10) at 11 zoos (58% of the zoos returning survey responses). All respondents reported a context for the target call that was about one or a combination of the following: food, keeper-gorilla interactions (e.g., training sessions), and/or the gorillas being interested in something and/or seeking/wanting attention.

We followed up with 11 zoos with requests for vocalization recordings when one or both of the following occurred: (1) at least one keeper confirmed the presence of the target vocalization in their gorilla group but did not supply a recording of the call or (2) at least one keeper provided a qualitative description of an attention-getting vocalization that we interpreted to be a potential description for the target vocalization (e.g., “soft barking,” “short, dry cough,” “huff noise”). Between our solicitations as well as the recordings already provided by some respondents with the original surveys, we received a total of 17 videos and/or audio clips of different gorillas from 11 facilities. Among these, and after selecting recordings of sufficiently high quality (n = 15), we confirmed the use of the target call by 6 gorillas (5 females and 1 male) housed at 4 different zoos. One of these gorillas (Macy) was also the subject of our experimental study at Zoo Atlanta, suggesting long-term use of the target call even after transferring between institutions.

## Discussion

In this study we explored whether zoo-housed western gorillas selectively used species-typical and/or species-atypical vocal signals to attract the attention of humans. We repeated an experiment known to elicit attention getting signals in great apes [46] and found that gorillas at Zoo Atlanta produced vocalizations and gestures significantly more often when human and food were present together than when either stimulus was presented alone. This was also true for a novel vocalization not previously described in the species repertoire and produced mostly during the keeper-food condition, which we named the attention-getting sound (AG) or “snough”. We then showed that the AG call was acoustically distinct from other gorilla calls produced in the same context: the food call or hum and the single grunt [63, 64]. Moreover, the results of the zookeeper survey suggest that the AG call is not idiosyncratic to the Zoo Atlanta gorillas, as survey respondents attributed its use to as many as 33 gorillas housed at 11 different zoos across the US and Canada. By examining video footage of 45% of these gorillas (15/33), we confirmed that the AG call was in fact produced by at least 6 gorillas (5 females and 1 male) housed at 4 different facilities. These results demonstrate that gorillas can modify their calls to produce a novel sound and furthermore confirm that they can produce their calls and gestures intentionally to modify the attention status of their caregivers.

Western gorillas at Zoo Atlanta use diverse signals to attract human attention, including vocalization and auditory/non-auditory gestures, with soft vocalizations of low frequency, banging or hand clapping, and shaking/waving a body part being some of the most common signals used. Although our sample size was small (n = 8 gorillas), all three communicative signals tested (i.e., the AG call, other vocalizations, and gestures) followed a similar pattern: they were all more frequently produced during the *keeper-food* condition compared to when either stimulus was presented alone. Regarding the gestures, contrary to our expectation, gorillas produced both auditory and non-auditory gestures more often in the third condition, possibly because the keeper position (at 90° instead of 180°) permitted the gorillas to see one of the keeper eyes, which might have led to the use of silent gestures to attract their attention. Among the vocalizations produced during the experiment, the most common call type was the novel attention-getting sound (85%), even if only half of the subjects used it (n = 4). The other call types (grunts and hums) were rare and in fact were never produced during the *food-only* or *keeper-only* conditions. Thus, in contrast to chimpanzees (see [75]), gorillas did not produce significantly more food calls (or hums: [63, 64]) when food was presented alone, indicating differing call functions between the two species. While chimpanzees produce food calls upon initial discovery to recruit specific individuals to the food source [76], western gorillas produce them only during food consumption [63, 64], potentially to coordinate spatial distances and/or reduce aggression between group members while feeding. However, since our study subjects were separated from other adults and were not provided food during the experiment, these potential food call motivations were not present. This may explain why we recorded only two hums throughout all trials.

The novel call type, the AG sound, has not previously been described in the repertoire of wild gorillas [63, 77–79]. Indeed, our study results indicate that it is used specifically to attract the attention of humans, suggesting that gorillas, as other apes, are able to produce novel sounds when encountering novel contexts (e.g., the chimpanzee raspberry: [59]; orangutan whistling: [54, 55]). However, because the vocal behavior of wild western gorillas (the gorilla species found in US and Canadian zoos) has only recently been described [63, 66], we cannot exclude the possibility that future studies may document the target call in wild populations, as well. Nevertheless, in our study, the AG call was never used by captive gorillas when communicating with one other (J. Carrigan pers. Comm.; R. Salmi unpublished data), supporting the idea that it is a novel sound not part of the typical gorilla-gorilla communication repertoire and that it emerged to address the communicative need of attracting human attention in captive settings. This, however, does not exclude the possibility that the same call may be separately innovated and used for different purposes in wild populations. The AG sound, in fact, is not the first vocal invention noted in gorillas. Perlman and Clark (57) attributed several novel utterances to the female gorilla Koko during interactions with caregivers. These included a fake cough/sneeze, which was accompanied by a hand gesture and an open mouth and strongly resembled our study’s AG sound, and a raspberry, used by Koko to obtain/request nuts and produced by lingual-labial fricative (i.e., folding her tongue length-wise, pressing it between her lips, and blowing). Indeed, across our survey, 11 participants representing 5 zoos reported raspberries as a common attention-getting strategy exhibited by the gorillas in their care, although no further details on how the gorillas articulated them were provided. The same call was recently described as potential vocal tradition in some wild populations of the congeneric mountain gorillas, though individuals there used it in a different context and with a different function [80]. Thus, vocal inventions and traditions, although rare, are present in the genus *Gorilla* and, in some cases, are shared by all great apes [81, 82].

The AG call is acoustically different to other gorilla calls commonly used during feeding (namely, hums and grunts), being of shorter duration and/or higher frequency yet still within the known vocal range of this species (R. Salmi pers. comm.). At Zoo Atlanta, the novel AG call was produced alone or in a series of calls by four gorilla females, equal to only 50% of our sample. Our analyses of video/audio recordings of potential samples of the AG call from gorillas at other zoos, however, yielded further insight: (1) it confirmed the AG call’s presence outside of Zoo Atlanta, (2) it provided evidence that the call is used by both sexes, and (3) it indicated that the number of gorillas to which survey respondents had attributed the AG call was most probably an overestimation. Though respondents attributed the target call to at least 33 gorillas, the number might be closer to 13 individuals (40%; considering the proportion of gorillas producing the target call among those for whom we received good quality recordings: 6/15). Misclassification of similar-sounding gorilla calls by the survey respondents was expected, particularly since the vocal repertoire of gorillas is highly graded [83] with no clear boundaries between call types and since call identification by ear, without visual inspections of spectrograms, is prone to error.

Although confirmed in some zoo gorilla populations in the US and Canada, the AG call is likely not as common as the more prominent raspberry call used by captive chimpanzees [62], which may indicate that zoo gorillas only recently adopted this sound for the purpose of getting the attention of humans. Moreover, the call may spread more slowly than the chimpanzee raspberry owing to the lower intensity of affiliative interactions [84], smaller social groups [85], and smaller captive population sizes of gorillas when compared to chimpanzees (~ 400 vs. ~1600 in 2019, North America; [74, 86]).

Since the presence of the AG sound is sparse even within institutions, social learning and independent innovation as opposed to genetic or ecological factors may better explain its distribution, similar to other ape calls that emerged in captive settings (chimpanzees: [60–62]; orangutans: [55]). The novel AG call and its usage could be learned during early ontogenetic developmental stages by those gorillas exposed to the signal, which could explain why most of the individuals using the AG call at Zoo Atlanta were related to one other, either vertically (mother-daughter) or horizontally (sister-sister). Among the 6 gorillas confirmed to use the call outside Zoo Atlanta, two females belong to the same group and are distantly related, two adult females are not related but are part of the same social group, the other two gorillas lived in different zoos, with one being Macy, who transferred from Zoo Atlanta after participating in our study. Although the use of the same call by gorillas within and outside Zoo Atlanta for the same purpose suggests transmission via social learning and/or parallel independent innovations, our study does not explain how the innovation/s occurred. Whether the AG (or snough) call has emerged randomly or has been learnt/modelled by observing humans, as seems to be the case for Koko’ fake cough and the orangutans’ whistle [55], remains unknown. We can only speculate that a sound somewhere between a cough and a sneeze would instinctively attract the attention of caregivers, whose responsibilities include the daily monitoring of their gorillas’ health. This in turn could explain independent innovation of the same call at different institutions where learning did not play a role in its spread. Future studies are needed to quantitatively assess the spread of the AG call and to determine its origin and transmission patterns across the captive gorilla population. Moreover, because the current study is unable to confirm whether the novel AG is a case of vocal usage learning (i.e., the use of an existing call-type in a novel context) or vocal adjustment learning (i.e., the modulation of an existing call-type) [87], future studies should also compare it to the entire vocal repertoire of captive gorillas.

Although, the vocal repertoires of primates, including those of apes, are characterized by innate calls at the species and possibly the genus level [88], our research contributes to a small but growing body of knowledge showing that primates do modify their vocal output based on experience [81], making them limited or moderate learners [18, 32]. The ability to generate novel acoustic signals represents an important characteristic of human language [89] with potentially early evolutionary roots, since all great ape species have been shown to generate novel sounds, although rarely, when encountering novel environments with novel communicative needs (i.e., captivity) (chimpanzee: [59]; orangutan: [54, 55]; bonobo; [82, 90]; gorilla: [57, this study]). This ability may not be limited to great apes, since examples of vocal accommodations to ecological and social contexts [e.g., 25, 91], the use of putative distinct vocal signals from populations of the same species [92], combinatory ability of vocal signals [e.g., 93–96], and the influence of parental vocal responses on the vocal development of infants [marmosets: 3], are some of the examples suggesting learning processes in the production of monkey calls as well. We thus support a paradigm shift in the study of vocal learning that (1) overcomes the dichotomous classification of vocal learners vs. non-vocal learners based on vocal mimicry alone and (2) embraces the multidimensionality of this complex continuum trait [17, 18, 20, 32, 97] to better explore the mechanisms involved in vocal learning [27] and the factors influencing its evolution.

## Supporting information

S1 Appendix Summary of the questionnaire distributed to survey respondents at AZA institutions. (DOCX)

S1 File Pairwise comparison between call types. (DOCX)

S1 Video Video of the novel attention getting call (Sukari–Zoo Atlanta). (MOV)

S1 Data Acoustic data used in discriminant function analysis. (CSV)
