# Supplementary material for: A novel attention-getting vocalization in zoo-housed western gorillas
Source: PLoS One. 2022 Aug 10;17(8):e0271871. doi: 10.1371/journal.pone.0271871 (PMC9365142; doi:10.1371/journal.pone.0271871)
Supplement: S1 Appendix — (DOCX) [file pone.0271871.s001.docx]

**Appendix A**

Summary of the questionnaire distributed to survey respondents at AZA institutions.

| 1 | Name of the zoo where respondent works |
| --- | --- |
| 2 | Three most common methods used by the gorillas to get the respondent’s attention (whether auditory, tactile, or visual); for vocal strategies, we requested further description in subsequent questions |
| 3 | If applicable, descriptions of any vocalizations reported in question 2 |
| 4 | If applicable, audio or video file(s) of any vocalizations reported in question 2 |
| 5 | Here, we shared a video of the present study’s target vocalization to determine if any gorillas in the respondent’s care exhibited this particular call |
| 6 | If applicable, the context in which any gorillas exhibited the target vocalization |
| 7 | If applicable, how many individual gorillas exhibited the target vocalization |
| 8 | If applicable, the names of any gorillas who exhibited the target vocalization |
| 9 | If applicable, the ages of any gorillas who exhibited the target vocalization |
| 10 | If applicable, the sexes of any gorillas who exhibited the target vocalization |
| 11 | If applicable, whether the target vocalization was ever used exclusively between conspecifics |
| 12 | If applicable, the context for a “yes” to question 11 |
| 13 | Any additional pertinent information |
| 14 | Consent for acknowledgment in the study |
