# Supplementary material for: A novel attention-getting vocalization in zoo-housed western gorillas
Source: PLoS One. 2022 Aug 10;17(8):e0271871. doi: 10.1371/journal.pone.0271871 (PMC9365142; doi:10.1371/journal.pone.0271871)
Supplement: S1 File — (DOCX) [file pone.0271871.s002.docx]

**Supplemental Information 3. Salmi et al. – Novel Attention Getting Call in Captive Gorillas**

**Table SI3.1** shows pairwise comparison results for duration (A), the mean frequency of the 2^nd^ dfa (B), and the minimum difference in frequency between 1^st^ and 2^nd^ dominant frequency bands.

**A**

| **Duration - Pairwise Contrasts** | | | | | | | |
| --- | --- | --- | --- | --- | --- | --- | --- |
| Call Type Pairwise Contrasts | Contrast Estimate | Std. Error | t | df | Adj. Sig. | 95% Confidence Interval | |
|  |  |  |  |  |  | Lower | Upper |
| AG - HUM | -2150.896 | 126.309 | -17.029 | 61 | .000 | -2461.844 | -1839.948 |
| AG - GR | -413.962 | 148.828 | -2.781 | 42 | .008 | -714.386 | -113.539 |
| HUM - AG | 2150.896 | 126.309 | 17.029 | 61 | .000 | 1839.948 | 2461.844 |
| HUM - GR | 1736.934 | 147.748 | 11.756 | 54 | 4.441E-16 | 1396.253 | 2077.615 |
| GR - AG | 413.962 | 148.828 | 2.781 | 42 | .008 | 113.539 | 714.386 |
| GR - HUM | -1736.934 | 147.748 | -11.756 | 54 | 4.441E-16 | -2077.615 | -1396.253 |

**B**

| **Q2 mean - Pairwise Contrasts** | | | | | | | |
| --- | --- | --- | --- | --- | --- | --- | --- |
| Call Type Pairwise Contrasts | Contrast Estimate | Std. Error | t | df | Adj. Sig. | 95% Confidence Interval | |
|  |  |  |  |  |  | Lower | Upper |
| AG - HUM | 648.400 | 85.148 | 7.615 | 60 | 6.777E-10 | 438.669 | 858.131 |
| AG - GR | 559.237 | 104.274 | 5.363 | 56 | 3.153E-6 | 319.124 | 799.351 |
| HUM - AG | -648.400 | 85.148 | -7.615 | 60 | 6.777E-10 | -858.131 | -438.669 |
| HUM - GR | -89.162 | 101.833 | -.876 | 60 | .385 | -292.850 | 114.525 |
| GR - AG | -559.237 | 104.274 | -5.363 | 56 | 3.153E-6 | -799.351 | -319.124 |
| GR - HUM | 89.162 | 101.833 | .876 | 60 | .385 | -114.525 | 292.850 |

**C**

| **Diffmean - Pairwise Contrasts** | | | | | | | |
| --- | --- | --- | --- | --- | --- | --- | --- |
| Call Type Pairwise Contrasts | Contrast Estimate | Std. Error | t | df | Adj. Sig. | 95% Confidence Interval | |
|  |  |  |  |  |  | Lower | Upper |
| AG - HUM | -47.477 | 41.916 | -1.133 | 61 | .262 | -131.300 | 36.345 |
| AG - GR | -213.154 | 50.173 | -4.248 | 47 | .000 | -337.685 | -88.624 |
| HUM - AG | 47.477 | 41.916 | 1.133 | 61 | .262 | -36.345 | 131.300 |
| HUM - GR | -165.677 | 49.457 | -3.350 | 57 | .003 | -279.554 | -51.801 |
| GR - AG | 213.154 | 50.173 | 4.248 | 47 | .000 | 88.624 | 337.685 |
| GR - HUM | 165.677 | 49.457 | 3.350 | 57 | .003 | 51.801 | 279.554 |
| The sequential Bonferroni adjusted significance level is .05.  Confidence interval bounds are approximate. | | | | | | | |

**Table SI3.2.** Number of calls for each call type (AG, HUM, BV) for each caller used in the Discriminant Function Analysis and permuted Discriminant Function Analysis. * indicates the gorillas that participated to the experiment portion of the study.

| **Caller** | **AG** | **HUM** | **BV** |
| --- | --- | --- | --- |
| Sukari * | 18 | 1 | 1 |
| Kuchi * | 3 | 5 | 6 |
| Chamba | 0 | 0 | 5 |
| Kudzoo * | 7 | 6 | 0 |
| Choomba * | 0 | 0 | 3 |
| Lulu * | 0 | 4 | 1 |
| Macy * | 3 | 1 | 0 |
| ***Total*** | ***31*** | ***16*** | ***16*** |
